# Measuring integrated HIV DNA ex vivo and in vitro provides insights about how reservoirs are formed and maintained

**DOI:** 10.1186/s12977-018-0396-3

**Published:** 2018-02-17

**Authors:** Marilia Rita Pinzone, Una O’Doherty

**Affiliations:** Department of Pathology and Laboratory Medicine, University of Pennsylvania, Philadelphia, PA USA

**Keywords:** ART, HIV, Integrated HIV DNA, Proviral DNA, Reservoir, Sequencing

## Abstract

The identification of the most appropriate marker to measure reservoir size has been a great challenge for the HIV field. Quantitative viral outgrowth assay (QVOA), the reference standard to quantify the amount of replication-competent virus, has several limitations, as it is laborious, expensive, and unable to robustly reactivate every single integrated provirus. PCR-based assays have been developed as an easier, cheaper and less error-prone alternative to QVOA, but also have limitations. Historically, measuring integrated HIV DNA has provided insights about how reservoirs are formed and maintained. In the 1990s, measuring integrated HIV DNA was instrumental in understanding that a subset of resting CD4 T cells containing integrated HIV DNA were the major source of replication-competent virus. Follow-up studies have further characterized the phenotype of these cells containing integrated HIV DNA, as well as shown the correlation between the integration levels and clinical parameters, such as duration of infection, CD4 count and viral load. Integrated HIV DNA correlates with total HIV measures and with QVOA. The integration assay has several limitations. First, it largely overestimates the reservoir size, as both defective and replication-competent proviruses are detected. Since defective proviruses are the majority in patients on ART, it follows that the number of proviruses capable of reactivating and releasing new virions is significantly smaller than the number of integrated proviruses. Second, in patients on ART clonal expansion could theoretically lead to the preferential amplification of proviruses close to an *Alu* sequence though longitudinal studies have not captured this effect. Proviral sequencing combined with integration measures is probably the best estimate of reservoir size, but it is expensive, time-consuming and requires considerable bioinformatics expertise. All these reasons limit its use on a large scale. Herein, we review the utility of measuring HIV integration and suggest combining it with sequencing and total HIV measurements can provide insights that underlie reservoir maintenance.

## Background

The introduction of combination antiretroviral therapy (ART) has profoundly modified the history of human immunodeficiency virus (HIV) infection. The majority of patients on ART have undetectable viral loads and life expectancy close to the general population [1–3]. Unfortunately, ART is not curative, and in the majority of individuals HIV viral load promptly rebounds after ART cessation. This is due to the presence of long-lived viral reservoirs, containing replication-competent proviruses, which currently represent the barrier to any curative approach [4–6]. “Shock and kill” strategies rely on the activation and immune clearance of viral reservoirs. The evaluation of efficacy of such interventions requires the accurate measure of the individual viral reservoir.

Measuring HIV reservoirs has been challenging. Historically, quantitative viral outgrowth assay (QVOA) has been considered the reference standard to measure the fraction of the HIV reservoir that is replication-competent [7]. Polymerase chain reaction (PCR)-based assays, such as total and integrated HIV DNA, have represented a cheaper, less time-consuming and less error-prone approach to study the reservoir, but have their own shortcomings [8, 9].

In this review, we summarize the technical and clinical strengths, as well as the weaknesses of measuring integrated HIV DNA. We also discuss the scenarios where, despite its limitations, integrated HIV DNA can still provide useful information, especially when combined with other techniques, such as proviral sequencing.

## The challenge of measuring HIV reservoir size

Measuring integrated HIV DNA has been instrumental in increasing our understanding of HIV biology. In the 1990s, Siliciano’s group published the first ground-breaking studies showing that resting CD4 T cells containing integrated HIV DNA were the main reservoir in patients on ART [10, 11]. The authors showed that replication-competent virus could be induced in vitro from resting CD4 T cells of patients with undetectable viremia by using QVOA. Initially, it was thought that latently infected cells form when HIV integrates in activated cells just before they return to a resting state [10–15]. However, additional studies demonstrated that resting CD4 T cells can be directly infected with HIV with delayed kinetics [16–25].

Historically, QVOA was extremely important because it captured a relevant attribute of the reservoir—that cells persisted without making any virus unless they were stimulated and then could produce virus. This was conceptually important because it explained why the reservoir was resistant to therapy. The assay relies on the purification of large numbers of resting CD4 T cells usually by negative selection, which are cultured in the presence of target cells to amplify released virions and activators to stimulate infected cells to release virions. QVOA requires a large amount of blood (~200 ml), or a leukapheresis product, in order to obtain the required number of resting CD4 T cells. QVOA is based on the limiting dilution method and the results are typically expressed as infectious units per million cells (IUPM) [7]. QVOA as currently performed is an underestimate of reservoir size as it is difficult to stimulate every replication-competent provirus. In fact, repeated stimulation of initially negative wells leads to reactivation of proviruses that were not induced in the previous round of stimulation [26]. This could be due to the stochastic nature of HIV reactivation [27]. Notably, repeated rounds of T cell stimulation can reactivate many of the latent proviruses that are resistant to expression. Proviral sequencing suggests the reservoir may be 6-fold larger than QVOA estimates [26]. Proviral sequencing studies have further called into question the value of QVOA as more intact proviruses were identified in effector memory (TEM) > transitional memory (TTM) > naive > central memory (TCM) T cells [28], while QVOA suggested that TCM contained the largest fraction of replication-competent proviruses [29].

After ART interruption virological rebound always occurs even when the reservoir is extremely small, as shown by the Mississippi baby [30] and the Boston patients [31, 32]. “Undetectable” HIV in these publications indicates not detected in a large volume of blood (typically ~180 ml or ~20–50 million CD4s). These patients could now be described as having reservoirs below a certain detection limit, such as < 1 infectious unit per 50 million CD4s. QVOA is not appropriate to detect small changes in the size of the reservoir that may occur in pilot clinical trials because of its limited reproducibility, the large number of patient’s cells, the expense, the technical expertise, and the significant labor required [33]. Considering these limitations, PCR-based methods were developed to provide upper limit estimates of HIV reservoirs, as an easier, cheaper and less error-prone tool that might compliment QVOA.

In the following paragraphs, we describe some scenarios where integrated HIV DNA provided unique insights about reservoir characterization, in settings where other assays could not be fully exploited because of the presence of unintegrated HIV DNA, ongoing replication (untreated infection, episodes of viremia on ART) or because of limited cell availability (studies on HIV persistence in cellular subsets).

### Integrated HIV DNA in cellular subsets

In the last 20 years, the HIV field has progressively gained a better understanding of the cellular subsets contributing to the reservoir size. Ostrowski et al. [15] showed that memory CD4 T cells contain 16-fold more integrated HIV DNA than naive cells consistent with the idea that the memory CD4 T cells make up the largest portion of the HIV reservoir. However, the difference between memory and naive cells (defined as CD62L + CD45RA + cells) was much smaller in patients infected with C-X-C chemokine receptor type 4 (CXCR4) viruses. This could be explained by the near absence of C–C chemokine receptor type 5 (CCR5) and high levels of CXCR4 in naive cells. Similarly, Chomont et al. [34] showed that the pool of cells containing integrated HIV DNA is mostly represented by cells with a memory phenotype. Measurements of integration provided important evidence suggesting that naive T cells contribute to the reservoir, which were then confirmed in a small subset of patients by QVOA as well [15, 34, 35]. Given the long intermitotic half-life of naive T cells, this subset may prove to be a significant under-investigated barrier to cure and integration measurements remain the primary evidence of their contribution to the reservoir. Notably, this data should be evaluated in light of recent studies on T memory stem cells (TSCM) [36–39], which are phenotypically similar to naive T cells, but can be distinguished by the expression of CD95 and interleukin 2 receptor subunit beta. Considering the long half-life of naive and TSCM, both cell subsets could be significant contributors to the reservoir.

Central memory (TCM, CD45RA-CCR7+CD27+) and transitional memory (TTM, CD45RA-CCR7-CD27+) CD4 T cells contain the majority of integrated HIV DNA, and could be responsible for reservoir maintenance/replenishment through several mechanisms, including antigen-driven and homeostatic proliferation. TCM were reported to be the main reservoir in immunological responders and individuals who started treatment early. On the other end of spectrum, in patients with low CD4 T cell counts the majority of HIV DNA was harboured by TTM. These cells have increased proliferative activity in comparison to TCM and may therefore contribute to the stability of the reservoir. The reservoir size was smaller in individuals with higher CD4 nadir, higher absolute CD4 counts and a CD4/CD8>1. Moreover, integrated HIV levels were significantly lower in patients who had started ART within the first year of infection [34].

Recent studies have provided an in-depth phenotypical analysis of cellular subsets that can be enriched for HIV DNA. Gosselin et al. [40] sorted blood memory cells according to the expression of CCR6, CCR4 and CXCR3 to differentiate the following subsets: T helper (Th)17 (CCR4 + CCR6+), Th2 (CCR4 + CCR6−), Th1Th17 (CXCR3 + CCR6+), and Th1 (CXCR3 + CCR6−). These subsets showed different susceptibility to HIV infection in vitro: in fact, cells with a Th17 and Th1Th17 profile appeared highly permissive to R5 and X4 HIV infection, whereas those with a Th2 profile were susceptible to X4 HIV replication only, and cells with a Th1 profile were relatively resistant to both R5 and X4 HIV replication. There was an enrichment for integrated HIV DNA in circulating CCR6 + T cells of HIV-infected subjects, both off and on ART, but a parallel depletion of these cells in comparison to uninfected controls, suggesting they may be preferentially infected and killed by HIV. Since the CCR6/C–C motif ligand-20 (CCL20) axis is important for mucosal homeostasis, more CCR6 + cells can potentially be recruited in tissues, such as the gut, the vagina and the brain, attracting additional susceptible cells at the sites of viral replication. The same group showed more recently that CCR6 + cells are enriched in the colon of individuals on ART in comparison to blood. Moreover, in both compartments CCR6 + cells harbour higher levels of total HIV DNA in comparison to CCR6- cells [41]. An enrichment for integrated DNA in CXCR3 + CCR6+ cells has been reported by others [42].

Immune checkpoint molecules are co-inhibitory receptors, physiologically involved in the containment of immune activation. Overexpression of several immune checkpoint molecules has been associated with T cell exhaustion and dysfunction. A recent study evaluated their association with HIV reservoir size [43]. In patients on stable ART, none of the markers alone was associated with integrated HIV DNA when adjusting for current CD4 count. However, the co-expression of Lymphocyte activation gene-3 (LAG-3), T cell Immunoglobulin and ITIM domain (TIGIT), and Programmed death-1 (PD-1) correlated with the frequency of cells harbouring integrated HIV DNA, after adjusting for nadir and current CD4 T cell count (*p* = 0.038). Memory CD4 T cells showed a gradual enrichment for integrated HIV DNA when expressing an increasing number of immune checkpoint molecules. Cells expressing the 3 markers were eightfold enriched for integrated HIV DNA in comparison to the whole CD4 population. The authors speculated that cells expressing these markers can be preferentially infected with HIV, or they may preferentially persist in comparison to the negative ones.

### Dynamics of integrated HIV DNA in acute and chronic HIV infection

 The first hint that treating patients early would be more effective at reducing reservoir size came from Strain et al. [44]. They showed that after one year of ART replication-competent HIV could not be detected by QVOA in any of the individuals starting ART during primary HIV infection (PHI) and in the majority of patients initiating therapy within 6 months after seroconversion.

Recent studies of the dynamics of integrated HIV DNA provide some clues to possible mechanisms behind restriction of reservoir size with early treatment including immune clearance. Both animal and human models have shown that HIV seeding occurs very early during HIV infection [45–47]. However, there is evidence that the earlier ART is started during acute infection the smaller the HIV reservoir is after virological suppression [48]. An in-depth study of acutely infected subjects in Thailand evaluated the dynamics of total, 2-Long Terminal Repeat (LTR) and integrated HIV DNA in untreated and treated acute HIV infection [49]. In untreated patients (Fiebig stage I/II (HIV RNA+, p24 ±, HIV IgM−)), integrated HIV DNA peaked at week 2 after enrollment, declined significantly between week 2 and week 6, and then gradually increased over time. By the end of the observation period (week 144), integration levels were significantly higher than at nadir (*p* = 0.02). Total HIV DNA did not capture this effect, likely because of the excess of unintegrated DNA: it increased rapidly, peaking at week 2, but did not change significantly afterwards in the untreated group. Treated individuals started ART immediately after enrollment (46% in Fiebig stage I/II). Integrated HIV DNA was 25-fold lower at week 2 and 100-fold lower at week 144 in comparison to untreated individuals. These findings have important clinical implications, since both total and integrated HIV DNA measures correlate with immune reconstitution, with immune activation and predict the time to viral rebound after ART interruption [50–53]. Thus, in certain settings integrated HIV DNA can be a correlate of reservoir size, despite the fact that it is an overestimate and has additional limitations discussed below.

Several studies have shown that initiating ART during acute infection is associated with a greater decline in the levels of integrated HIV DNA [54–56]. A limitation of these studies is their small size, but the consistent finding from all three groups that integrated HIV DNA was cleared more rapidly and effectively if patients were treated early makes these results more convincing. Murray et al. [56] showed the decline of integrated HIV DNA was biphasic and that the first phase of decay was significantly faster when patients were treated early after HIV infection with a half-life of 10 versus 43 days for the first phase of decay (*p* = 0.04) and then 63 versus 172 days. Meanwhile the rate of decay for total HIV was similar for both groups. Pinzone et al. showed that acutely infected individuals exhibited a significant drop in integrated HIV levels 12 months after ART initiation, while integration levels barely changed in patients treated during chronic infection [54], consistent with Koelsch et al. [55]. Moreover, Buzon et al. [57] found treating the earliest stages Fiebig III/IV resulted in a larger drop in integrated HIV DNA than treating in Fiebig V; uniquely, the decline in integrated HIV continued in those patients treated at the very earliest stages over several years. Pre-ART integration levels also correlated with viral load (r = 0.86) and negatively correlated with CD4/CD8 ratio (r = − 0.52), consistent with the idea that integrated HIV DNA can provide a surrogate marker for reservoir size [54] and consistent with [34, 56, 58]. These longitudinal studies highlight that integrated HIV DNA measures provide different and complementary information to total HIV DNA when an excess of unintegrated HIV DNA may exist.

Potential reasons for lower reservoirs when initiating ART early include (1) less escape from cytotoxic T lymphocyte (CTL) [59, 60], (2) more functional CTL during acute infection [59, 61, 62], (3) preferential protection of TCM [50, 63] and (4) increased susceptibility to ART. The latter possibility would seem likely if the fraction of HIV harbouring replication-competent proviruses is larger in acute infection. A limitation of these studies is the lack of information on the fraction of proviruses that are replication-competent. If replication-competent proviruses plateau early after infection, then the reduction in integration levels observed in acute infection is likely to reflect the effectiveness of ART against replication-competent proviruses while in individuals with chronic infection the majority of proviruses are defective and only a small fraction of them will be cleared by antiretroviral drugs. If replication-competent proviruses continue to accrue at a constant rate, this would suggest that the immune system is more effective early after HIV infection. Bruner et al. [64] have recently provided the first attempt to characterize the proviral landscape by sequencing proviruses during acute infection. The authors showed that defective proviruses accumulate early in HIV infection, making up over 93% of the proviral pool, even when ART is started within the first 2–3 weeks from enrolment. Alternatively, it is possible that a significant fraction of the reservoir is expressed and potentially cleared even in individuals treated during chronic infection, but the clonal expansion of defective clones may mask a drop in reservoir size by DNA measures [65]. Sequencing proviruses at multiple time points could provide new insights on the dynamics of intact/defective proviruses over time.

### Longitudinal studies show integrated HIV DNA increases over time

In the absence of ART, integrated HIV DNA accumulates over time after a brief decline that may be immune-mediated [49, 54]. Pinzone et al. [54] monitored longitudinally integrated HIV DNA in 6 individuals followed from acute to chronic infection (mean observation time 6 years), showing that integrated HIV DNA increased progressively over time (from 109 to 1941 copies/million peripheral blood mononuclear cells (PBMCs)). The authors compared the increase in reservoir size observed in chronic progressors (CPs) versus long-term nonprogressors (LTNPs). As expected [57, 66], they found that LTNPs have much lower integrated HIV DNA levels. However, in the absence of ART LTNPs experienced an increase in the levels of integrated HIV DNA over time (from 17 to 34 copies/million PBMCs over 5 years), consistent with evidence of ongoing replication [67–69]. Integration levels did not significantly change in ART controls. Among the chronic progressors, the rate of integration increase varied greatly; in fact, two patients showed some decline in integrated HIV DNA within the first 2 years of observation, followed by an increase in integration levels, suggesting that some transient immune control existed early during infection. The different accumulation rate observed in LTNPs and chronic individuals can be due to differences in CTL functions. However, loss of CTL function over time did not explain the increase in integrated HIV DNA in LTNP patients as CTL function did not decline over time. We speculate that reservoir expansion could be due to ongoing viral replication in sanctuary sites, such as the B-cell follicles, where CD8 T cells are functionally excluded [70–72]. The increase in integrated HIV DNA over time suggests that the true reservoir size increases over time [8, 32].

### Structured Treatment Interruptions and integrated HIV DNA measures

Several studies have evaluated the changes in total HIV DNA levels after ART interruption [52, 53, 73, 74], but few have addressed changes in integrated HIV DNA [53, 75]. The VISCONTI cohort provides an example of enhanced frequency of functional cure for HIV, as a higher fraction of individuals who were started on ART within 2 months of infection were able to maintain undetectable viral loads for several years after ART withdrawal [50]. In the Spartac study and the ANRS 116 SALTO study, total HIV DNA levels were shown to be predictive of the timing of viral rebound in patients treated early after infection [52, 53]. Azzoni et al. showed in a small pilot study of patients on ART who received treatment intensification with pegylated interferon alpha-2a (IFN-α-2a) that integrated HIV DNA actually declined after treatment interruption in the subset of patients that maintained virologic control [51]. More data on the kinetics of integration levels coupled with proviral sequencing after STI would improve our understanding of reservoir expansion in this setting.

### Integrated HIV DNA and reservoir clearance

Integrated HIV DNA can be an useful tool to assess CTL-mediated clearance of infected CD4 T cells [76]. Graf et al. measured the levels of integrated and 2-LTR intermediates in CD4 T cells from LTNPs that had been superinfected in vitro and cocultured with autologous CD8 T cells. They showed the preferential clearance of integrated over 2-LTR DNA in the presence of CTL. This was consistent with the hypothesis that Gag+ cells are preferentially cleared, since integrated HIV but not 2-LTR expresses Gag in an efficient manner under short-term coculture [77]. The authors also found that integrated HIV DNA inversely correlated with CTL capability to clear infected cells both in LTNPs and CPs. These findings again are consistent with the idea that CTL activity controls the expansion of HIV reservoirs and at least in the very early stages of infection immune clearance plays a role in limiting reservoir size.

Integration measures have helped capture the possible role of immune clearance in reservoir formation and maintenance. In fact, in the setting of untreated infection other assays, such as total HIV DNA or QVOA, cannot be used to assess the dynamics of reservoir change over time. In the aforementioned study from the acute Thailand cohort [49] the drop in integrated HIV DNA between week 2 and 6 suggests clearance, possibly immune-mediated, of infected cells. Similarly, in the study from Buzon et al. [57] patients starting therapy in the earliest Fiebig stages did have smaller reservoirs. In [54], some chronic progressors did show an initial contraction of the reservoir during the acute phase of infection, followed by reservoir expansion, suggestive of initial immune control, which was then lost over time.

### Integrated HIV DNA in studies using latency reversal agents (LRAs)

A few trials have evaluated the change in integrated HIV DNA levels after the administration of LRAs to disrupt latency, such as vorinostat [78], panobinostat [79], and romidepsin [80]. Interestingly, in none of these studies a significant change in integration levels was found at a cohort level. This could be due to the fact that only a minority of patients may respond to the intervention and the change in their reservoir size can be masked when looking at the average response in the cohort. Moreover, at an individual level, defective proviruses that contain no open reading frames (ORFs) would not be cleared by eradication strategies, and if such proviruses were prominent they would mask clearance in the population of intact proviruses. Since some studies only target one ORF (HIV Gag in the Vacc4x study) [80], only intact proviruses and proviruses expressing Gag would be expected to be cleared with this approach, resulting in only small changes in HIV integration (in most cases < twofold). Notably, one patient in Vacc4x study did show a reduction in integrated and total HIV DNA and QVOA, and may represent a responder [80]. Follow-up studies sequencing proviruses in this potential responder may clarify if the patient is a true responder. One potential advantage of measuring integrated HIV DNA is that the error of the assay is low and this makes it possible to identify a small reduction in individual responders by monitoring the patients longitudinally. While total HIV DNA measurements also have a small error, we speculate many therapeutic approaches, especially LRAs, have the potential to induce a round of reverse transcription (unpublished data). In this case, total HIV DNA might not capture a reduction in the size of the reservoir, which could be detected by integrated HIV DNA [81].

### Combined use of HIV DNA intermediates to model the dynamics of reservoir over time

In some studies, mathematical modelling has provided important insights into how different HIV intermediates in resting and activated cells change over time on ART. Murray et al. [58] analyzed longitudinally the dynamics of HIV intermediates in resting and activated cells of 8 patients with acute infection and 8 patients with chronic HIV starting an antiretroviral regimen containing raltegravir.

Before ART initiation, resting cells had the highest levels of 2-LTR and 2-LTR/integrated HIV DNA ratio. These observations are consistent with direct infection of resting cells in vivo [16–25], as supported by recent modelling [82]. 2-LTR would be expected to accumulate in resting cells as a consequence of the longer life span of resting cells as well as less efficient integration in resting cells [18, 19, 83].

Interestingly, after 1 year of ART, the levels of total, integrated and 2-LTR DNA were similar in resting and activated cells. This has important implications for eradication studies. At first blush, we would expect HIV DNA levels to quickly decline in activated cells after starting ART, if ongoing replication were stopped [84], as a result of several mechanisms, including cell death due to viral cytotoxicity. However, the persistence of HIV DNA in activated T cells suggest that cells may be converting from a resting to an activated phenotype and vice versa. This, in turn, suggests that activation of an HIV infected cells does not always result in cell death before the cell can return to a resting state. This, in turn, suggests the basic idea of “shock and kill” may be more difficult to achieve than initially thought since cell activation from latency may not result in cell death.

## Integrated HIV DNA: technical aspects

### Principles of the assay

HIV integration is measured using a nested real-time approach [85, 86]. The first step of PCR anchors a forward primer to the human *Alu* element and a reverse primer to the HIV genome. *Alu* is a repeat element in the human genome that occurs approximately every 3,000 base pairs. Only integrated HIV DNA is exponentially amplified by the first step, whereas unintegrated HIV DNA is linearly amplified by the HIV primer since only one strand can be copied. The second step is a real-time PCR approach within the HIV LTR. In order to adjust for the amount of unintegrated HIV DNA that can be linearly amplified, during the first step some wells contain only the HIV-specific primer. This controls for the background signal coming from unintegrated HIV and is used to define a threshold for a signal that represents a positive well for integration. *Alu*-HIV PCR is the most applied method to measure integrated HIV DNA. Less common methods include inverse PCR, linker ligation PCR and gel separation [10, 87, 88].

In the gel separation method, DNA samples are run on a gel to separate genomic high-molecular weight DNA from episomal DNA. The genomic DNA recovered from the gel is then used to measure HIV DNA by PCR. Recently, Lada et al. used pulse-field gel electrophoresis combined with droplet digital PCR, and showed good correlation with *Alu*-HIV PCR (r = 0.7, *p* = 0.023), and efficient removal of unintegrated forms, but low yields from the gel (on average 21%) [87].

### Choice of the PCR primers

Different labs measuring integrated HIV DNA use different HIV primers for the first amplification step. O’Doherty’s lab uses a primer located in a conserved region of the Gag gene (primer SK431). Chomont’s lab uses a primer annealing in the U3-R junction of the LTR [89]. The difference in primers used for the first step has important implications, as in the first case only proviruses containing an intact Gag region will be amplified, while with the second primer all the proviruses with an intact LTR will likely be amplified, including a larger number of massively deleted proviruses. One primer may have advantages over the other, depending on the specific experimental question that is being asked. For example, in studies evaluating reservoir clearance after priming for Gag CTL, the Gag primer may be preferred, as in that case integrated HIV decline may represent a surrogate of reservoir contraction. On the other hand, the *Alu*-LTR assay will capture all the integrated HIV DNA and therefore provides increased sensitivity over *Alu*-Gag. This could be an important advantage in the assessment of reservoir changes after therapeutic interventions (for instance bone marrow transplantation), when the levels of residual HIV DNA are expected to be extremely low and a very sensitive assay is required to detect any residual HIV.

### Quality control for robust measurements

Consistency of amplification is affected by variations in master mixes, Taq polymerase, as well as variability between thermocyclers. Large volume PCR master mixtures minimize systematic variation. An integration standard can be included in all runs to test thermocycler conformity from run to run and to identify PCR inhibition (by adding the standard to patient samples) [9, 85]. Some laboratories utilize serial dilutions of cell lines (e.g. ACH-2) to create a standard curve to quantify integrated HIV DNA [89]. The ACH-2 cells are not entirely transcriptionally silent and contain variable numbers of HIV integrations (from 5 to 10 in our hands) [90]. Every lab should verify the number of proviruses per cell in a given lot of ACH-2 cells before using them as a standard in these assays. This is actually an advantage for the ACH-2 cells line, as it has sufficient diversity of integration sites to roughly capture the diversity of distances to *Alu* present in acute infection and can be used to estimate integration frequency while other cell lines with 1–2 proviruses do not provide strong estimates.

For each infected cell, the distance between the integrated provirus and the nearest *Alu* element is variable. Therefore, each provirus will be amplified at different efficiency depending on its distance from the closest *Alu* [17]. This represents an important limitation of the assay, which is mitigated by repetitive sampling. Moreover, to reduce variability between runs as well as variability between different laboratories, our laboratory currently measures integrated HIV DNA using the Poisson distribution. This allows the quantification of integrated HIV DNA without using a standard curve. We target 30–80% of positive wells at two dilutions in a 96-well plate to obtain the most robust result, as error increases outside of this range. This implies that we need ~500 proviruses per patient to obtain a robust measure of the integration levels [unpublished data]. It follows that the number of cells required for the assay will largely vary depending on the individual integration levels. The chance that the well contains no integrated HIV DNA (negative reaction) or 1 or more proviruses (positive reaction) will follow the Poisson distribution. The number of copies of integrated HIV can be calculated from the frequency of positive wells by PCR without the need of a standard curve [91], though we do apply a correction factor as our assay detects ~10% of integrations [91] (20% of integrations are detected with recent improvements due to decreased gag background).

Measuring integrated HIV DNA robustly in LTNPs is challenging. It requires a large number of cells, as in some patients the integration level may be as low as 1–5 copies/million PBMCs, which can be a limitation if apheresis products are not available [66]. To increase the sensitivity of the assay, a large number of cells per well are needed, and this requires best-quality DNA in order to avoid PCR inhibition.

Some labs compensate for limited numbers of available cells by testing large numbers of patients [89]. However, a low number of repeats reduces the sensitivity of the assay, which implies that negative results should be interpreted carefully, as they may reflect the limited quantity of cells tested.

## Measuring integrated HIV DNA: a summary of pros and cons

### Strengths

On a technical level, integrated HIV DNA is relatively inexpensive, robust, and potentially high-throughput in comparison to QVOA. Total and integrated HIV DNA can be combined to capture ongoing replication. A full review of total HIV DNA as a measure of reservoir size is provided in another chapter of this special issue [92]. In patients on long-term ART, integration levels are relatively similar to total HIV DNA and consistent with a relatively stable reservoir [34, 81]. Total and integrated HIV DNA provide different insights [93]. Total DNA showed a similar decline in acute and chronic infection with a sevenfold decline in the first year and a slower decline over the next few years from pre-ART levels [93]. In contrast, there was a tenfold decline of integrated HIV DNA levels in acute infection, whereas there was only a twofold decline in patients treated chronically [54].

Mexas et al. [81] showed the utility of combining total and integrated HIV DNA in clinical trials. In the presence of detectable viremia, the authors showed an increase in the ratio between total and integrated HIV. Moreover, they evaluated the change in reservoir size of patients on stable ART who received IFN-α-2a + ART for 5 weeks, followed by IFN-α-2a alone for 12 weeks. 45% of patients maintained a viral load < 400 copies/ml during ART interruption and were considered “responders”. Treatment with IFN-α-2a led to an increase in total over integrated HIV DNA as well as an increase in viremia on ART and after ART interruption, suggesting that IFN-α-2a treatment induced ongoing replication. In responders, the administration of IFN-α-2a also led to a decrease in integrated but not total HIV DNA levels. This discrepancy between total and integrated HIV DNA could be due to the imbalance between the immune-mediated clearance of cells containing integrated HIV (reduction in integration levels) and de novo infection of new cells (increase in total DNA). These results suggest that the concomitant use of total and integrated HIV DNA can provide insights into the changes in reservoir size after therapeutic interventions.

Moreover, in some cases total HIV DNA cannot be used to measure reservoir size. In most patients off ART unintegrated HIV represents the most abundant form. In those cases, total HIV DNA measures would be largely driven by variable levels of linear and circular non-integrated forms. Therefore, integrated HIV DNA can represent a more appropriate tool to measure reservoir size in patients off ART.

Integrated HIV DNA is a robust assay, and can capture smaller changes than the QVOA assay. Integration levels correlated with QVOA in a comparative study of reservoir assays [33] (r = 0.7, *p* = 0.0008). In this study, QVOA did not correlate with total HIV DNA, probably because of data censoring; a few samples were negative for total HIV DNA by digital droplet PCR, thus reducing the strength of the correlation. Similarly, Mendoza et al. [94] reported that QVOA correlated with integrated HIV DNA in a cohort of LTNPs (r = 0.72, *p* = 0.03). More recently, similar findings were published by Kiselinova et al. [95] in a cohort of 25 long-term treated patients who started ART during chronic infection. The authors found that integrated HIV DNA correlated with total HIV DNA (R^2^ = 0.85, *p* < 0.001) and QVOA (R^2^ = 0.44, *p* = 0.041). Thus, while integration is an overestimate of reservoir size and while the number of defective proviruses vary between patients, in some settings measuring integrated HIV DNA can serve as a less error-prone surrogate of reservoir size.

### Weaknesses: variable overestimate of reservoir size

Most of the integrated HIV DNA is not replication-competent, as it contains large deletions, mutations originating from viral reverse transcriptase or from innate host defense mechanisms (e.g. APOBEC3G). PCR-based methods overestimate reservoir size as the majority of proviruses are defective in individuals on ART [26, 96]. Those proviruses will not be distinguished from replication-competent ones using *Alu*-HIV assays. Table 1 provides three possible outcomes of eradication trials when using integrated HIV DNA to assess if a therapy is effective. In scenario 1, an intervention might be effective in reducing the “real” reservoir, but have no effect on defective proviruses such that integrated HIV DNA would remain unchanged. This might occur if clearance of the infected cells required virion release or if a strategy required high-level expression of Gag, which would require in turn expression of Tat and Rev; thus, these proviruses are generally largely intact and unlikely to be defective. In scenario 2, an intervention that targets only defective proviruses would decrease the levels of integrated HIV DNA, but this drop would not reflect a decrease in the size of the “true” reservoir. This might occur if replication-competent proviruses are more resistant to transcription or translation than defective ones. possibly due to the repressive nature of the site of integration. In scenario 3, a decline in integration would likely capture a reduction in reservoir size if an intervention targets both defective and replication-competent proviruses, though the reduction would not likely capture the precise change in the true reservoir as defective and replication-competent proviruses are not expected to be targeted proportionally. This could occur if an immune therapy can clear both defective and replication-competent proviruses that are capable of expressing HIV proteins as was proposed to occur in [51, 81]. If transcription of replication-competent proviruses is not repressed more than transcription of defective proviruses, the immune response should be more effective at clearing replication-competent proviruses that defective ones, since replication-competent proviruses have 9 ORFs for the immune system to target. The previously mentioned IFN-α-2a trial suggested this third scenario could occur. Given that IFN-α-2a would likely increase immune clearance of all protein-expressing cells, it was likely that defective proviruses with intact ORFs as well as intact ones could both be cleared. Notably proviruses that contain no ORFs should not be cleared, though these represent a minority of proviruses [64, 97].

**Table 1:** Possible outcomes of eradication trials when using integrated HIV DNA to assess the change in reservoir size

Possible outcome in eradication studies	How does the outcome affect reservoir measures?
1. Preferential reduction in intact proviruses	Integrated HV DNA is likely to be unchanged. Proviral sequencing would likely detect a reduction in the fraction of replication-competent proviruses which could be combined with integration measures to determine the absolute reduction in intact proviruses
2. Preferential reduction in defective proviruses	Integrated HIV DNA would decrease, without reflecting a contraction in the size of the “true” reservoir. Proviral sequencing would likely capture the change in the fraction of defective proviruses
3. Decrease in both intact and defective proviruses	If intact and defective proviruses are targeted differently, a decrease in integrated HIV DNA would occur, but would not likely be proportional to the decrease in the number of intact proviruses. Proviral sequencing combined with integration measures would likely capture the changes in the reservoir size and character

HIV integrates preferentially within regions of active transcription [98, 99]. *Alu* repeats are also more prominent in gene-rich regions. As described, the integration standard was designed to correct for the tendency of HIV to integrate closer to *Alu* sites. However, this correction did not account for clonally expanded integration sites. With time on ART clonal expansion occurs [100] and there appears to be selection with a tendency for clones that are near cell cycle genes. In fact, it has been shown that after several years on ART more than 40% of proviruses are located in the genome of cells that have undergone clonal expansion after HIV integration. Clonal expansion may result from selection of proviruses integrated HIV preferential selection into genes promoting cell growth, as recently shown by Maldarelli et al. [101]. These genes also tend to be close to *Alu* sites. As a consequence, proviruses that are closer to *Alu* sequences are likely to be preferentially expanded over time on ART. Thus, the presence of clonal expansion can result in apparently higher levels of integrated HIV DNA over time in comparison to total HIV DNA measures. Integration site analysis of patients with discrepant total and integration measurements may clarify why integration levels can appear to be slightly higher in some patients on ART. While clonal expansion is an appealing explanation for discrepancies between total and integrated HIV DNA, in our hands integrated and total HIV DNA are relatively constant over time on ART which is not consistent with this explanation. Regardless, the exact level is less important than the relative change for revealing reservoir expansion, contraction and ongoing replication.

There are some instances where knowing the exact level is important as well, for instance to estimate the total-body reservoir size. One scenario could be represented by STI after bone marrow transplantation, when the residual reservoir size is expected to be extremely low. In that case, the use of PCR assays, especially total HIV DNA, along with extensive sampling, likely represents the most sensitive tool to assess how much HIV persists in the body.

### Solutions to the hurdles involve combining integration measures with proviral sequencing

Combining integration measures with proviral sequencing to identify intact proviruses may represent the best tool to estimate the size of the HIV reservoir, but the assay is expensive and labor-intensive, and requires considerable bioinformatics expertise, limiting its scalability in large cohorts. As more data accumulate on reservoir growth and decay, it may be possible to choose cohorts with similar reservoir size and sequence characteristics, in which case PCR measures of integration might be useful to identify responders to a therapy, but accurate measurement of reservoir reduction would likely involve sequencing as well.

## Conclusions

 Measuring HIV reservoirs robustly is still a challenge for the field. Every available marker has its own strengths and weaknesses. The choice of the most appropriate marker(s) depends on the experimental question that is being asked. Measuring integrated HIV DNA has increased our understanding of HIV dynamics but, as discussed, the assay has several limitations, which impose a careful use of this tool in clinical studies. Proviral sequencing combined with integration measurements will likely provide the closest estimate of reservoir size, and the most powerful tool to characterize and monitor the proviral landscape in HIV-infected individuals.

## Abbreviations

ART: antiretroviral therapy
CXCR4: C-X-C chemokine receptor type 4
CCL20: C-C motif ligand 20
CCR5: C-C chemokine receptor type 5
CP: chronic progressor
CTL: cytotoxic T lymphocyte
CTLA-4: cytotoxic T-lymphocyte-associated protein-4
HIV: human immunodeficiency virus
IC: immune checkpoint molecule
IFN-α: interferon alpha
IUPM: infectious units per million cells
LAG-3: lymphocyte activation gene-3
LN: lymph node
LRA: latency reversal agent
LTNP: long-term nonprogressor
LTR: long terminal repeat
PBMC: peripheral blood mononuclear cell
PCR: polymerase chain reaction
PD-1: programmed death-1
PHI: primary HIV infection
ORF: open reading frame
STI: structured therapeutic interruption
TSCM: T memory stem cell
TCM: central memory T cell
TEM: effector memory T cell
Th: T helper
TIGIT: T cell Immunoglobulin and ITIM domain
TIM-3: T cell immunoglobulin-3
TTM: transitional memory T cell
QVOA: quantitative viral outgrowth assay
